# Machine Learning and Artificial Intelligence for Infectious Disease Surveillance, Diagnosis, and Prognosis

**DOI:** 10.3390/v17070882

**Published:** 2025-06-23

**Authors:** Brandon C. J. Cheah, Creuza Rachel Vicente, Kuan Rong Chan

**Affiliations:** 1 Program in Emerging Infectious Diseases, Duke-NUS Medical School, 8 College Road, Singapore 169857, Singapore; brandon.cj.cheah@u.nus.edu; 2 Departamento de Saúde Coletiva, Universidade Federal do Espírito Santo, Vitória 29090-040, Espírito Santo, Brazil; vicentecrachel@gmail.com

**Keywords:** machine learning, artificial intelligence, infectious diseases management, surveillance, diagnosis, prognosis

## Abstract

Advances in high-throughput technologies, digital phenotyping, and increased accessibility of publicly available datasets offer opportunities for big data to be applied in infectious disease surveillance, diagnosis, treatment, and outcome prediction. Artificial intelligence (AI) and machine learning (ML) have emerged as promising tools to analyze complex clinical and molecular data. However, it remains unclear which AI or ML models are most suitable for infectious disease management, as most existing studies use non-scoping literature reviews to recommend AI and ML models for data analysis. This scoping literature review thus examines the ML models and applications that are most relevant for infectious disease management, with a proposed actionable workflow for implementing ML models in clinical practice. We conducted a literature search on PubMed, Google Scholar, and ScienceDirect, including papers published in English between January 2020 and April 2024. Search keywords included AI, ML, public health, surveillance, diagnosis, prognosis, and infectious disease, to identify published studies using AI and ML in infectious disease management. Studies without public datasets or lacking descriptions of the ML models were excluded. This review included a total of 77 studies applied in surveillance, prognosis, and diagnosis. Different types of input data from infectious disease surveillance, clinical diagnosis, and prognosis required different ML and AI models to achieve the maximum performance in infectious disease management. Our findings highlight the potential of Explainable AI and ensemble learning models to be more broadly applicable in different aspects of infectious disease management, which can be integrated in clinical workflows to improve infectious disease surveillance, diagnosis, and prognosis. Explainable AI and ensemble learning models can be suitably used to achieve high accuracy in prediction. However, as most of the studies have not been validated in different cohorts, it remains unclear whether these ML models can be broadly applicable to different populations. Nonetheless, the findings encourage deploying ML and AI to complement clinicians and augment clinical decision-making.

## 1. Introduction

Despite growing attempts at the treatment and prevention of infectious diseases, Emerging Infectious Diseases claimed 13.9 million lives in 2019 [1]. The 2019 Global Burden of Disease study estimated that 49.0% (6.82 million) of all deaths caused by infectious diseases, including parasitic diseases and respiratory infections, stemmed from low- and middle-income countries (LMICs) [1,2]. Thereafter, the coronavirus disease-19 (COVID-19) pandemic resulted in an unprecedented spike in morbidity and mortality, particularly in LMICs, due to their limited healthcare infrastructure. Poor governance and the lack of funding for diagnostic tools are some of the reasons for the poor healthcare infrastructure in these countries [3]. Moreover, the higher presence of multimorbidity further complicates infectious disease management, increasing mortality [1,2,4]. Effective public health surveillance, diagnosis, prognosis, and implementation are thus critical for controlling infectious diseases in LMICs.

Data-driven diagnosis can enable greater precision of diagnosis, personalized treatment, and prediction of disease outcomes [5]. Recent years have witnessed substantial growth in big data applied in healthcare, with advancements in high-throughput technologies, digital phenotyping, increased availability and accessibility of publicly available datasets, better data storage, innovative methods for data collection, and overall heightened data consciousness. However, the data generated can be from diverse sources, including electronic hospital patient records, omics output [6], phylogeny trees [7], deep sequencing data [6], images [8], biomarker tests [9], or web surveillance data [10] on newly emerging clusters. Furthermore, heterogeneous patient presentation, varied disease manifestations, multi-morbidities, and host variation can add to the complexity of infectious disease management and impair clinical diagnosis [11]. Therefore, a significant challenge will be to analyze and interpret the data, to identify the most important measurements and features that are meaningful and useful for clinical decision-making.

Recent developments in Artificial Intelligence (AI) have shown promise in organizing large datasets, reducing dimensionality, and extracting relevant features for analysis [5]. Machine learning (ML), a branch of AI, offers significant potential for analyzing multimodal data for infectious disease control [12]. The field of applied ML has increasingly been focused on developing more accurate and sophisticated models. However, with the increase in the number of ML models, it can be challenging to identify the most suitable ML algorithms for data analysis. While the more refined models may accurately predict the training data, some of these models may result in overfitting, limiting the generalizability of such models for new data prediction [13].

Most of the existing reviews on AI and ML in infectious diseases focus on isolated aspects of infectious disease management, such as biopreparedness [14,15], diagnostics [16], prediction [17] or laboratory testing [18]. Some recent reviews have synthesized these aspects and explore potential clinical applications [19,20]. However, most of these studies were analyzed based on the literature reviews, which may introduce selection bias, as the results and recommendations were not interpreted based on comprehensive coverage of the existing literature. Additionally, many of these studies do not examine how AI and ML can be implemented and integrated in clinical settings [21] or focus only on specific diseases [22]. The aim of this scoping review is thus designed to comprehensively summarize the current literature on the applications on AI and ML in infectious disease surveillance, diagnosis, and prognosis. Moreover, based on these models, we also propose implementation strategies by proposing an actionable workflow for implementing AI and ML models in infectious disease clinical practice. We believe that these considerations will provide a foundation for integrating ML and AI in infectious disease management.

The main objectives of this scoping review are thus to: (1) Evaluate the types of AI and ML models that are most appropriate for infectious disease management; (2) Determine the tools and applications that can be used for the development of AI and ML for clinical applications; and (3) Provide an overview on the translatability and potential of AI and ML applications in infectious disease for healthcare professionals through a tutorial-styled review. A scoping review was conducted to identify the most relevant AI and ML models used in infectious disease management, emphasizing applications in surveillance, diagnosis, and prognosis. In addition, we consolidated the performance of the different AI and ML models in the various infectious disease applications, highlighting their applicability and limitations for infectious disease management. Available models are stratified by applications to processes in the patient trajectory. Finally, a summary and workflow for AI and ML models in infectious disease management is provided. The review does not focus on the technical details of each model, but rather provides a broad comparison for potential use cases that can be developed further in clinical settings. Our review indicated the applicability of Explainable AI and ensemble learning models for infectious disease surveillance, disease diagnosis, and prognosis, as many of these models display high accuracy in prediction. These studies raise the exciting possibility of using these models to develop AI and ML in infectious disease management.

## 2. Methods

### 2.1. Information Sources and Search Strategy

We reviewed the literature available in PubMed, Google Scholar, and ScienceDirect. Google Scholar was chosen to identify the gray literature that may not be indexed in other repositories. Other databases, such as Web of Science and Embase, were excluded due to issues in institutional access. The search keywords used were ‘AI’, ‘ML’, ‘Public Health’, ‘Surveillance’, ‘Diagnosis’, ‘Prognosis’, and ‘Infectious Disease’. The Boolean Operators ‘OR’ and ‘AND’ were used in tandem with wild-card placeholders (*) to factor in possible word combinations. For example, ‘Diagnos*’ would include words like ‘Diagnosis’, ‘Diagnostics’, and ‘Diagnoses’. Only title and abstracts were searched. The complete search terms used were (((((AI[Title/Abstract]) OR (ML[Title/Abstract]) AND (Infectious Disease[Title/Abstract])) AND (Diagnos*[Title/Abstract])) OR (Public Health[Title/Abstract])) OR (Surveillance[Title/Abstract])) OR (Prognosis[Title/Abstract]).

### 2.2. Selection and Data Collection Process

We included the literature published from 1 January 2020 to 9 April 2024. Literature types included journal articles, books and their sub-chapters, conference proceedings, peer-reviewed commentary papers, and perspective articles. Records were identified through Google Scholar, PubMed, and ScienceDirect. The original database search resulted in 1150 records from Google Scholar, 2038 from PubMed, and 9316 from ScienceDirect. From the records, 2867 were related to public health and surveillance, 6492 were related to clinical diagnosis, and 3145 were related to clinical prognosis. After removing duplicates, the remaining 9630 records were screened. The exclusion criteria were (1) articles not written in English; (2) bot-generated articles; (3) duplicate records; (4) non-peer-reviewed articles or preprints; (5) non-relevant articles; (6) articles addressing ‘AI’ and ‘ML’ with no concrete evidence of techniques or algorithms that could be verified; (7) inconclusive articles; and (8) articles with conclusions not directly supported by publicly available datasets.

## 3. Results

### 3.1. Systematic Search for AI and ML Models Used in Infectious Disease Management

**Figure 1:**
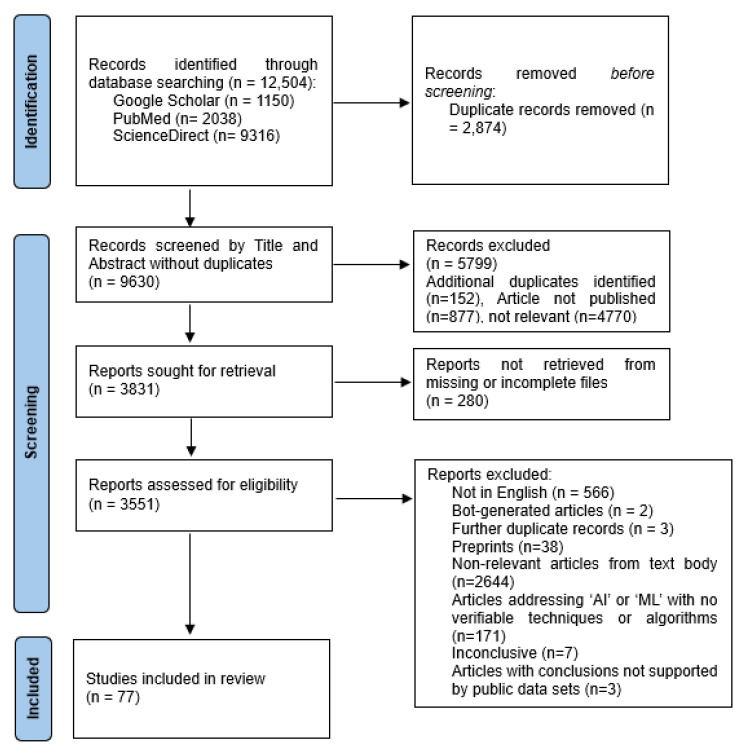
Flow chart of search strategy employed to identify machine learning models used in infectious disease management. AI, Artificial Intelligence; ML, machine learning.

**Figure 2:**
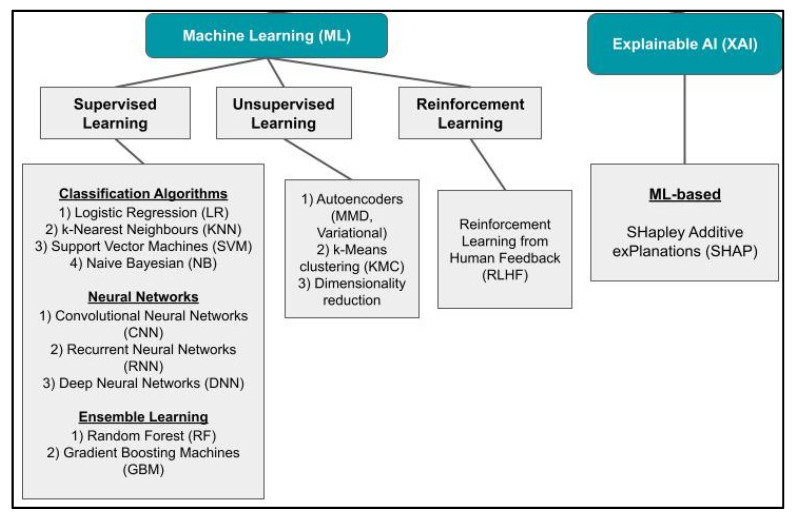
Overview of AI and ML techniques used in infectious disease management, classified into supervised learning, unsupervised learning, reinforcement learning, and Explainable AI.

As this study’s objective was to summarize the ML and AI models applied in infectious disease management, we organized the data into three broad application categories: surveillance, prognosis, and diagnosis. The models were evaluated based on the metrics of accuracy, precision/positive predictive value, recall/sensitivity, specificity. Additionally, F1-Score, area under the receiver operating characteristic curve, and Matthew’s correlation coefficient metrics were used. Database search and screening were primarily performed by Brandon Chong Joon Cheah (B.C.J.C.), and the outputs were validated by Kuan Rong Chan (K.R.C.). After removing the duplicate records, irrelevant articles not related to applications of ML and AI to infectious disease management, and research articles that did not indicate how the ML and AI methods were performed (Figure 1), 77 studies were included in the scoping review, of which 18 were related to public health and surveillance, 36 to diagnosis, and 23 to prognosis. As part of the inclusion criteria, articles selected were screened to have undergone a peer-review. Figure 1 shows the flow of study identification and selection. We identified ML and AI applications used in surveillance, prognosis, and diagnosis, which included supervised, unsupervised, and reinforcement learning ML models (Figure 2). Metrics to evaluate AI and ML model performance are consolidated in Table 1, while the performance of the ML and AI models used in infectious disease management are summarized and presented in the results in Table 2, Table 3 and Table 4.

### 3.2. Supervised, Unsupervised, and Reinforcement ML Models Used in Infectious Disease Management

ML models can be generally classified into the categories of supervised, unsupervised and reinforcement learning. Supervised ML entails mapping between a set of input variables (x) and an output variable (y) [13]. Such a mapping process would be most beneficial when the output is known, and when relationships between variables are well-understood. By predefining the classes for comparison, supervised ML strives to determine the parameters and measurements that best separate the different classes. For instance, given the research aim of identifying blood biomarkers to predict the risk of a patient progressing to a severe disease, supervised ML can assist in the comparison of severe and mild disease patient subgroups to achieve this aim. The most common model types used can be generally classified as decision trees, logistic regression, neural networks, and ensemble learning (Figure 2). The model types have different approaches to labeling and stratifying the data, each with advantages and limitations. Decision trees and logistic regression offer interpretable conclusions with limited scalability to large sets of data [23]. Neural networks offer efficient scalability with increasing amounts of data but are deficient in interpretability due to hidden network layers that are difficult to interpret even for model developers, which may lead to overfitting of data [24]. Ensemble learning methods, on the other hand, combine several predictions from multiple techniques to achieve a better predictive result [25,26]. However, these models are generally more complicated, requiring more computing resources for training and deployment.

Supervised approaches are favored as investigators can have better control over the comparisons to be made, which can provide insights with fewer sample sizes. The training procedure is usually more straightforward because the outcome is already pre-defined. However, as most of the supervised models are based on comparisons between distinct categorical data, labeling errors and potential confounders from other external variables, such as subject demographics, comorbidities, and treatment, may influence the results, thereby affecting the accuracy, sensitivity, and specificity of the model. For instance, the parameters and measurements involved in the prognosis and diagnosis of severe dengue can vary between the young and the elderly [27], so the classification of severe and mild dengue patients may require further stratification of the data into young and elderly patients to gain insights into the measurements that are influenced by age.

Unsupervised ML tries to discover patterns and propose structures of unlabeled data to circumvent the limitations of pre-assigning data labels [28]. It is generally more complex and typically strives to reduce the dimensionality of the data to assign clusters for further analysis. Some examples include principal component analysis, hierarchical clustering, k-means clustering, and weighted gene co-expression network analysis. Unsupervised ML will typically require large training datasets to identify trends and patterns, achievable only if a systematic framework is in place to actively collect relevant data. The unsupervised approach is preferred when classifying data into pre-defined clusters is not straightforward or when such classifications are impossible. For instance, unsupervised ML methods can potentially help detect or predict novel infectious disease outbreaks in clinical settings, which cannot be performed using supervised ML methods.

Reinforcement ML relies on a punishment–reward system, where the machine chooses an outcome that maximizes the rewards based on observed states and actions taken [29]. Diseases requiring multiple stages of decision-making from clinicians work better with Reinforcement ML methods [29]. Finally, Explainable AI (XAI) works to provide interpretability to ML models by delivering accompanying evidence or reasons for decision outcomes and processes [30]. A commonly used XAI feature is SHapley Additive exPlanations (SHAP), which uses concepts from cooperative game theory to gauge the impact of individual features on the prediction made by an ML model [31,32]. XAI addresses the ‘black box’ nature of ML due to the lack of human-interpretable values, which can now be resolved by providing values of feature importance to rank each feature’s contribution to a predicted outcome. The utility of SHAP comes from its model agnostic nature, which ensures the applicability of the XAI regardless of the choice of model [33], which may be more versatile than other XAI models, like Gradient-weighted Class Activation Mapping (Grad-CAM) used in Convolutional Neural Networks (CNNs) [34].

#### Methods Used to Evaluate AI and ML Model Performance

The performance of ML models in prediction can be measured using different metrics. Table 1 provides an overview of the commonly used metrics to assess performance.

**Table 1:** List of commonly chosen metrics used to measure machine learning model performance. Formula and a brief description of the different metric measurements that can be used to evaluate machine learning and Artificial Intelligence performance.

Metric	Calculation	Description
Positive Predictive Value (PPV)/ Precision	$\frac{TP}{TP+FP}$	Probability of the presence of disease given a positive test result [35]
Negative Predictive Value (NPV)	$\frac{TN}{TN+FN}$	Probability of the absence of disease given a negative test result [35]
Accuracy	$\frac{TP+TN}{TP+FP+TN+FN}$	Measurement of how well a model predicts the correct class or the fraction of predictions that the model correctly identified out of all the cases [36]
Sensitivity/ Recall	$\frac{TP}{TP+FN}$	Probability of a positive test result given the presence of disease [35,36]
Specificity	$\frac{TN}{TN+FP}$	Probability of all negative samples that are correctly predicted by the model [36]
AUROC	$\int_{0}^{1}\mathrm{ROC}$	The area under the graph of sensitivity against 1-specificity
F1-Score	$\frac{2\cdot Precision\cdot Recall}{Precision+Recall}$ $=\frac{2TP}{2TP+FP+FN}$	Weighted harmonic mean between precision and recall [36]
Matthew’s Correlation Coefficient (MCC)	$\frac{\left(TP\cdot TN\right)-(FP\cdot FN)}{\sqrt{\left(TP+FP\right)\cdot \left(TP+FN\right)\cdot (TN+FP)\cdot (TN+FN)}}$	Weighted classifier score factoring all four confusion matrix categories and imbalanced class data into account [36,37]

Accuracy is one of the most common metrics to evaluate model performance. A model with higher accuracy values is generally considered a more robust prediction model than a model with a lower accuracy. However, this interpretation may be incorrect for two reasons. Firstly, the accuracy metric is skewed by class imbalance. In a binary classification problem, the accuracy metric weights the individual class ratio proportionally to the class size [38]. When a majority class size is large, the probability of the ML classifier choosing that class is significantly higher, resulting in a disregard for the minority class [39]. In an extreme example, consider a series of Computed Tomography (CT) scans of dengue patients; if 99% of the images are of dengue-negative cases, the ML classifier can achieve 99% accuracy by merely blind guessing all the patients to be negative. Secondly, an overly high accuracy value can imply algorithm overfitting, limiting the model’s ability to generalize and make predictions in future testing sets [40]. Decision trees and neural networks are generally more prone to overfitting, which can provide an unrealistically high score of accuracy on the training set but low accuracy on future test sets. Ensemble methods, such as Random Forest (RF), can reduce overfitting. Researchers can also consider stopping training early when the accuracy score no longer improves for neural networks to minimize overfitting [41]. These models’ accuracy can be validated in an independent investigation or on a different patient cohort to evaluate whether the model is overfitting.

Positive predictive values (PPVs) or negative predictive values (NPVs) can be alternatively used to measure performance, but these metrics should consider disease prevalence. Thus, PPVs and NPVs are best evaluated with other performance metrics. On the other hand, the Area Under Receiver Operating Characteristic (AUROC) can be more robust to overfitting than accuracy as it depicts a calibrated trade-off between sensitivity and specificity. Similarly, the F1-Score depicts the harmonic mean of precision and recall and can provide a more comprehensive evaluation of the model’s performance despite imbalanced datasets. Finally, to assess model performance across multiple classes, such as in disease and symptom profiles, Matthew’s Correlation Coefficient (MCC) can be used (Table 1) [42].

### 3.3. Applications of AI and ML in Infectious Disease Management

An effective integration of public health surveillance, diagnosis, and prognosis is critical for controlling infectious diseases. Given that most infectious diseases are diagnosed because of physiological abnormalities observed by clinicians [43], the current diagnosis and treatment solutions will largely depend on the clinician’s competencies and experience. However, over-reliance on individual clinician expertise can contribute to increased workload and potentially impact clinical judgment. In such cases, data-driven diagnosis and prognosis can help reduce the burden of over-reliance on individual clinician expertise, providing more standardized clinical care and assessment to patients. Based on our literature search, we identified that the processes that have the potential for optimization by ML include surveillance, diagnosis, prognosis, and treatment [44].

In a non-crisis scenario, a comprehensive clinical history can be taken to ascertain the details of the patient’s symptoms, forming the Chief Complaint (CC). Besides the temporal profiling of the symptoms to understand the disease trajectory, details on the symptom manifestation, previous infection, and disease severity can be useful parameters for clinical diagnosis. Collecting additional patient data on the symptoms and demographics can allow data stratification for more precise diagnosis and prognosis.

Based on the disease and symptom profile and kinetics, the clinician can make an initial diagnosis, which can then be confirmed by a laboratory Polymerase Chain Reaction (PCR) test. The clinician can leverage clinical parameters or biomarkers to facilitate patient triaging after knowing the causative agent that resulted in disease manifestation by 24–48 h post-CC. Patients deemed having a low risk of severe disease progression would be discharged, whereas patients with high risk of progression would be hospitalized.

The ideal non-crisis scenario, where measurements for patient demographics, history, and detailed blood tests can be taken for each patient, is not likely to hold true in an ongoing infectious disease crisis. In a crisis, an increased health burden on the hospitals due to a sharp increase in patient cases can result in a lower staff-to-patient ratio and increased Hospital Acquired Infections (HAIs), leading to higher severe disease and patient mortality [11,45].

**Figure 3:**
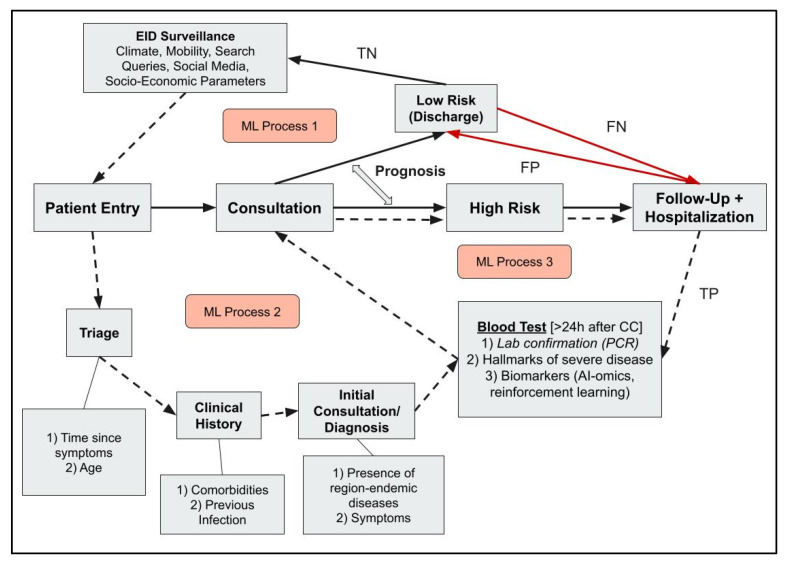
Flowchart demonstrating the patient trajectory of a typical infectious disease profile. ML can be potentially employed in surveillance (process 1), diagnosis (process 2), and prognosis (process 3) to facilitate and accelerate these processes, which will be critical particularly during a virus pandemic or epidemic. Solid lines represent the shortened patient trajectory in response to an infectious disease pandemic, which can be facilitated with the implementation of ML or AI; dotted lines represent an ideal patient trajectory, and host of potential parameters can be leveraged by ML; red lines represent potential treatment burdens on the hospital infrastructure during a pandemic. FN, False Negative; TN, True Negative; FP, False Positive; TP, True Positive; ML, machine learning; CC, Chief Complaint; PCR, Polymerase Chain Reaction; EIDs, Emerging Infectious Diseases.

Such a scenario follows a shortened clinical process, demonstrated by the solid lines in Figure 3. In the absence of a data-driven framework for clinical decisions, there will be a greater reliance and emphasis on clinician experience to determine the diagnosis and prognosis of the patient, which can be compounded by mental stress imposed because of an ongoing epidemic or pandemic. Every False-Positive (FP) or False-Negative (FN) case will exert a greater strain on hospital resources as they can hoard them from longer-than-needed stays in the hospital. On the other hand, FN patients who are mistakenly sent home may potentially progress to severe disease, which can be potentially life-threatening if timely interventions are not provided. Moreover, the FN patients may promote disease transmission if the pathogen is highly transmissible, which may cause further spread of the disease.

In our literature search, we identified three major processes that have the potential for ML intervention, shown in Figure 3. Process 1 includes public health and surveillance at the policymaking level. This provides contextual information on the disease and the pathogen emergence patterns governed by epidemiological conditions even before patient entry. For instance, it is well established in the literature that climate [44,46,47,48,49,50] and mobility [44,51,52] influence infectious disease incidence, especially for vector-borne diseases in LMICs. Warming of the climate, for example, results in increased vector competence, mosquito survival, biting rates, and viral replication [50].

Surveillance includes the timely risk assessment of an emerging infectious disease and warning public health officials early on the extent of disease transmission outside a given local community. Surveillance data encompass temporal and spatial components that exhibit significant non-linear patterns, which can be challenging to interpret using conventional threshold setting and statistical analysis methods [53]. ML can assist in integrating such multi-modal data and, when used correctly, even potentially provide a timely analysis to address the emergence of novel viral strains.

For example, in the recent pandemic caused by Severe Acute Respiratory Syndrome-Coronavirus 2 (SARS-CoV-2), fast-emerging viral strains have been known to be caused by the rapidly changing antigenicity of the spike protein and mutations in the Receptor Binding Domain (RBD), resulting in increased viral infectivity and communal transmission [54,55]. The existing method of identifying such mutations or new viral strains is mapping the genetic sequences into phylogenetic trees using alignment-based or alignment-free methods [56]. ML can, however, map structural protein regions into higher-dimensional space, allowing for visualization and analysis at a fraction of the computational cost and time, as shown in a SARS-CoV-2 study by Cahuantzi et al. [57]. Timely analysis of genetic data from variants of concern can benefit infectious disease surveillance, increasing the effectiveness of monitoring infectious clusters for disease control [57].

Process 2 involves triage and diagnosis, where an informed diagnosis is made after obtaining information from clinical history, symptoms, and blood tests. The role of ML is to integrate the data obtained from the triage process, demographic parameters, clinical history, and diagnosis to predict the risk classification of patient cases. The nature of retrospective data collection and posteriori knowledge generation is a data-driven process that provides an informed interpretation to the clinician to guide diagnosis. Dimensionality reduction in ML extracts key features relevant to the disease. Better algorithms in ML and deep learning result in a significant decrease in the complexity of analysis, reducing the running time and the need for manual programming to map clinical signs to diagnoses [19,58]. These processes facilitate streamlining workflow, reducing clinician error in decision-making.

Process 3 includes patient prognosis and ongoing infectious disease research. The presence of diagnostic tests and biomarker databases, such as the Infectious Disease Biomarker Database, can be leveraged by ML to inform clinicians and infectious disease researchers on the hallmarks of severe disease [9]. Patients who have poor disease prognosis leading to hospitalization can be potential data points for biomarker analysis. These data points can serve as TP for severe disease fed back into the integrated ML ecosystem. The ML process can serve as a feedback system to clinicians for future patient consultations, triggering the process of reinforcement learning. The back-and-forth iterative nature of continuous diagnosis and prognosis is essential, given that healthcare is increasingly moving toward personalized treatment [59].

The capability of ML intervention should be used to complement, not replace, clinicians and clinical decision-making. While it is natural to direct studies toward the comparison of clinician versus algorithm efficiencies, it would be more beneficial to study how an ML framework can be developed, applied, and improved to augment clinical decision-making [19]. Furthermore, ML should be seen only as a decision-support tool. The final responsibility for patient health outcomes and care must remain with clinicians to interpret ML outputs, which are most beneficial within the clinical context.

### 3.4. Roles of ML in Infectious Disease Public Health and Surveillance

One of the promising application areas for ML in infectious disease control is public health and disease surveillance. The types of public health and disease control are governed by the epidemiology of infectious diseases, which has been addressed by models such as the Eco-Epidemiology Triad model [60]. ML techniques developed in the literature are focused on evaluating the contribution of climate, mobility, Search Engine Queries (SEQ), social media, socioeconomic factors, and web-based surveillance on disease outbreaks [14,61,62,63,64,65,66,67,68,69,70,71,72,73,74,75,76,77,78,79,80]. These diverse data sources provide valuable insights into the dynamics of infectious diseases, which can be utilized for more effective predictive models to inform decision-making. However, clinical oversight remains critical despite the presence of diverse data sources and ever-increasing predictive power of the existing models to ensure patient safety and accountability.

#### 3.4.1. Climate

Several studies have demonstrated the effect of climate on infectious disease transmission, given that it influences local-scale disease dynamics and has implications for pathogen evolution [44]. Additionally, the researchers have employed ML techniques, including RFs, Support Vector Machines (SVMs), and Neural Networks, to uncover complex relationships between environmental factors and the transmission of infectious diseases [14,61]. Recurrent Neural Networks (RNNs), such as the Attention-Based Long-Short Term Memory Algorithm (LSTM-ATT), have demonstrated accuracy and specificity values of more than 0.8 using a 36-month lookback window in a forecasting study of dengue fever with climate data [81]. Another work on predicting a dengue outbreak in Malaysia had factored in humidity, rainfall, temperature, and windspeed data. The study found that the SVM with a Linear Kernel model demonstrated the highest accuracy and specificity of 70% and 95%, respectively, but with low sensitivity and precision values of 14% and 56%, respectively [62]. However, it should be noted that both studies isolated climate-related factors as a proxy for infectious disease transmission and did not include factors such as mobility and human behavior, potentially improving the prediction of viral outbreaks.

While the studies above have demonstrated climate’s impact on infectious disease transmission and the need for inclusion as a variable within ML models, climate has different impacts on different infectious diseases. For instance, warmer climates may have a greater impact on arbovirus transmission as they rely on mosquito vectors, which are more sensitive to temperature changes, as compared to diseases with human-to-human transmission, such as COVID-19. It then follows that climate-based models developed for use in arboviruses, such as dengue, may be less informative for COVID-19. Therefore, care must be taken to examine the individual disease transmission modes and pathogenesis before the inclusion of climate as a variable.

#### 3.4.2. Mobility

The relationship between mobility and the number of infectious disease cases, especially for SARS-CoV-2, has been identified as non-linear [63]. The non-linearity of the relationship is seldom addressed by traditional compartmental models, like the Susceptible–Exposed–Infected–Recovered (SEIR), due to its assumption of spatiotemporal homogeneity [64]. A study by Zhang et al. addressed the spatiotemporal propagation of the SARS-CoV-2 pandemic via RF and Logistic Regression (LR) algorithms, demonstrating that LR had a superior performance in Beijing and Guangzhou with AUROC values of 0.92 and 0.86, respectively [82]. Using LR, SVM, k-Nearest Neighbor (KNN), Multilayer Perceptron (MLP), RF, and eXtreme Gradient Boost (XGBoost), another study by Katragadda et al. determined that mobility is an essential factor in explaining an increase in SARS-CoV-2 case numbers. Notably, the study revealed that the impact of mobility is much more substantial in the initial phases of a pandemic than in the later phases [65]. This implies that the mobility feature in an integrated ML model can be reduced upon progressing further in the stage of a pandemic. For well-characterized infectious diseases, such as COVID-19, the existing data sources for mobility, such as SafeGraph, can be leveraged to provide state-level mobility data [65]. On the other hand, mobility data for tropical diseases are currently obtained through empirical proxies, such as mobile phone Call Detail Records obtained at a country level [66] or Google Mobility datasets. New datasets have been proposed to build upon the Google Mobility dataset upon its discontinuation in 2022 [83]. However, the lack of data on mobility appears to be the weakest link in adopting ML into infectious disease forecasting. Better methods and mobility assessment may thus be required to allow the collection of higher-quality data to develop better ML models for disease outbreak prediction.

#### 3.4.3. Search Engine Queries (SEQs) and Social Media

Another promising area where developments in ML in infectious disease public health are being seen is the application of Search Engine Queries (SEQs) and social media. The appearance of disease-related keywords in search engines or on social media platforms is likely correlated with infectious disease incidence. The correlation between health-seeking behavior and the early detection of infectious disease cases has been explored in multiple studies [67,68]. However, the utilization of ML for the analysis of SEQs or social media data has only surfaced recently with the advent of the SARS-CoV-2 pandemic. A study by Jang et al. utilized a Natural Language Processing (NLP) model built using an LSTM-RNN architecture named ‘Seq2Seq’ to determine positive case numbers in the pandemic. Importantly, the study determined that the positive correlation between the related SEQs and case numbers was not disease-specific and is potentially generalizable [69]. A follow-up work by the same group had added a word-embedding technique to the previously studied NLP model known as ‘Word2Vec’, which converts words to vectors to preserve semantic similarity. The study found an increase in the Pearson’s Correlation Coefficient (PCC) value from previous models to 0.21 with an RMSE of 0.08, indicating a higher correlation between COVID-19 outbreak data and Word2Vec Google Trends with a decreased error of prediction [70]. However, search entries are likely symptom-based unless a direct diagnostic measurement is readily available. Infectious diseases presenting similar symptoms may be confounding factors for semantic analysis alone. As such, SEQs should not be the only mode of warning for the presence of infectious diseases. Furthermore, PCC values do not provide a meaningful correlation and comparison with other standardized metrics, such as sensitivity and specificity. Reporting of metrics relevant for comparison can be included for clearer benchmarking.

Social media has surfaced as a low-cost, readily available data source for ML applications in infectious disease public health. A recent study by Kim and Ahn examined outbreak prediction using media articles with SVM, Deep Neural Networks (DNNs), and Semi-Supervised Learning methods. SSL demonstrated the best performance with accuracy, AUROC, and F1-Score values of 0.838, 0.791, and 0.832, respectively, for six months of training data [71]. The advantage of media analysis over SEQ analysis is the ability to obtain useful warning signs of an impending infectious disease outbreak in regions with low internet penetration, which is a pertinent issue in LMICs. Another important direction for ML applications is sentiment analysis in social media data. For instance, a systematic review by Gupta and Katarya showed that Twitter (now X) was the primary data source for 64% of the research related to health-related data collection [72]. However, as most of the evaluation is currently performed for COVID-19, it will be interesting to evaluate the contribution of SEQs and social media on other disease outbreaks.

#### 3.4.4. SocioEconomic Factors

Socioeconomic factors are key predictors of infectious disease burden, such as inadequate hygiene, access to treatments, and sanitation can promote pathogen disease transmission, such as dengue, Chikungunya, Zika, and Hepatitis A viruses [73,74,84,85]. Targeted intervention for these high-risk groups has been demonstrated to reduce mortality and morbidity rates in infectious disease patient cases [73]. The use of ML to uncover possible socioeconomic factors affecting infectious disease incidence is explored in a study by Kananura comparing Gradient Boosting Machines (GBMs), Least Absolute Shrinkage and Selection Operator (LASSO) regression, and LR. The GBM model demonstrated excellent promise in rural and urban settings for predicting Acute Respiratory Infection (ARI) cases in under-five-year-old Ugandan children with a testing set accuracy and AUROC of 0.96 and 1.00, respectively [75]. However, more research remains to be performed to validate this study’s exceptional metrics and explore innovative ways to measure the impact of socioeconomic factors in predicting infectious disease outbreaks.

Another similar study by Kalayou, Kassaw, and Shiferaw compares multiple ML techniques of RF, Decision Trees (DTs), SVM, NB, KNN, and XGBoost and ensemble learning models. The ensemble model demonstrated promise in classifying ARI cases in under-five-year-old Ethiopian children with an accuracy, sensitivity, and AUROC of 86%, 84.6%, and 0.87, respectively [76]. Importantly, the use of SHAP after analysis with ML determines feature importance, which allows the end user to retrospectively determine which features were assigned the highest weightage in the algorithm’s decision. The features deemed necessary by the study ranged from the type of household toilet used to the mother’s educational level [76].

Besides applying ML to individual parameters in infectious disease public health, incorporating a combination of socioeconomic variables with climate and mobility parameters to predict infectious disease outbreaks can provide additional insights into the critical contributors responsible for infectious disease outbreaks. For example, in a study by Sebastianelli et al., an ensemble model consisting of an algorithm combining GBM, LSTM, and SVM was used to predict dengue incidence in Brazil. The ensemble model averaged a normalized Root Mean Squared Error (RMSE) of 0.124 across all the studied districts. Interestingly, the model can also be extended to Peru, highlighting the importance of developing reproducible ML algorithms for surveillance and epidemiological studies [77].

#### 3.4.5. Web-Based Surveillance

The literature on applying ML to infectious disease surveillance has been centered around two core objectives—obtaining data from transnational databases and anomaly detection as a function of disease spread. Web-based surveillance systems, such as Google Flu Trends, ProMEDMail, and Medisys, remain to be the most widely used databases for infectious disease monitoring and data collection [78,79]. However, the failure to identify epidemiological signals from the lack of nationally coordinated algorithms and the underrepresentation of minorities is a significant barrier to effective disease surveillance [86]. A study by Kim et al. compared the CNN and bidirectional LSTM techniques in classifying documents obtained from web-based surveillance systems for outbreak prediction. The study distinguished between document and sentence learning levels, with the LSTM algorithm yielding an AUROC, accuracy, and F1-Score of 0.9547, 0.8817, and 0.8835, respectively [78].

On the other hand, ML algorithms have been developed to detect anomalies in disease surveillance data. A study by Eze et al. used a combination of a dimensionality reduction algorithm known as Principal Component Analysis and statistical methods, such as Minimum Covariance Determinant and Stochastic Outlier Selection, to detect disease anomalies in web-based surveillance data. Interestingly, the combination of three algorithms has been shown to cover 73.53% of all anomalies detected globally [80]. We believe that future improvements in ML-driven anomaly detections will potentially help quickly identify outbreaks caused by unknown pathogens with pandemic potential.

### 3.5. Roles of AI and ML Models in Diagnosis

ML in disease diagnosis has gained substantial traction in oncology, with direct applications in ovarian cancer [87,88,89,90], breast cancer [91], and lung cancer [92]. Moreover, the Food and Drug Administration (FDA) has approved DermaSensor, an AI-based handheld diagnostic device for skin cancer [93]. Given the pre-existing applications of AI and ML emerging technologies in oncology, applications into infectious diseases where image recognition is required—i.e., clinical image scans and diagnoses—can be investigated.

Given the time-sensitive nature of infectious disease presentation, an accurate and timely differential diagnosis is critical to improve disease outcomes and reduce community transmission. The misdiagnosis of infectious diseases with other febrile diseases can potentially have fatal consequences from administering inappropriate treatments [94]. The role of ML and AI is thus to improve the accuracy of diagnosis and reduce the influence of bias introduced by clinicians, which can lead to misdiagnoses. The most developed ML algorithms have been applied to classify images, clinical signs, and unstructured text classification.

#### 3.5.1. Imaging

A large proportion of studies have been dedicated to using ML-driven imaging approaches for the diagnosis of infectious diseases, including X-ray radiography [8,95,96,97,98,99,100,101,102,103,104], CT [8,104,105,106,107,108,109,110], photography [111,112,113], microscopy, and blood sera analyses [114,115,116,117]. Given that high-dimensional data with high numbers of features or attributes are contained in images collected in the studies, CNNs, DNNs, and their variants have become the model of choice [118]. These ML models have efficiently extracted the most critical features from images, allowing the better detection of anomalies and outlier features in images. Table 2 overviews the key studies utilizing ML-based imaging architectures for infectious disease diagnosis. The values listed in Table 2 are based on metrics of the best model identified in each study, with all studies reporting on test sets’ performance.

**Table 2:** Overview of studies using ML-based imaging architectures for infectious disease diagnosis. A dash in a cell refers to metrics that are unreported by the study. The best-performing architectures in each image category are in bold.

Image Category	Architecture	Accuracy	Precision/PPV	Recall/ Sensitivity	F1-Score	AUROC	Specificity	MCC	Reference
X-ray	DenseNet201	0.99	0.97	0.97	0.97	-	0.9895	-	[101]
	Custom CNN	0.9819	0.9767	0.9833	0.9733	-	-	-	[98]
	VGG19	0.9888	0.9870	0.9904	0.9987	0.9939	-	-	[99]
	Texture Extraction and SVM	0.9547	0.9471	0.9618	0.9544	-	0.9624	-	[96]
	EfficientNetB4 and ResNet50	0.92	0.97	0.92	0.94	0.90	-	-	[97]
	**Stacking NN and SVM**	**0.9962**	**0.9966**	**0.9962**	**0.9962**	-	-	-	[97]
	EfficientNet and ResNet	-	-	-	-	0.89	0.79	-	[100]
	Custom CNN	0.9872	0.9989	0.9966	0.9977	-	-	-	[102]
	GoogleNet and ResNet50	0.98	0.9471	0.9402	0.9389	-	0.9633	-	[103]
X-ray and CT	**Custom CNN**	**0.9940**	**0.9886**	**0.9941**	**0.9846**	-	-	-	[104]
	VGG19	0.9167	0.86	1	0.92	0.92	-	-	[8]
CT	VGG16	0.98	0.9799	0.9799	0.9799	0.9790	-	-	[109]
	AlexNet	0.9310	-	0.9180	-	0.9870	0.9460	-	[106]
	Inception-ResNetV2 and ResNet18 and Multi-Layer Perceptron	0.994	-	0.843	-	0.92	0.828	-	[107]
	ResNet34	0.9547	0.9947	0.9216	0.9567	0.9974	0.9942	-	[110]
	ResNet34	0.90	0.95	0.87	-	0.83	0.94	-	[105]
	**Custom CNN and Ensemble**	**0.9973**	**0.9946**	**1**	**0.9973**	**0.9973**	-	-	[108]
Photograph	ResNet50	0.8417	-	-	-	-	-	0.7715	[112]
	**MonkeyNet and Grad-CAM**	**0.9891**	**0.9892**	**0.9891**	**0.9891**	**0.9997**	-	-	[111]
	InceptionV3	0.94	-	0.88	-	-	1	-	[113]
Microscopy	YOLOv2 and ResNet50	-	0.7120	0.9190	-	-	0.8970	-	[117]
	Patch-U-Net	-	0.9380	0.8170	-	0.9740	-	-	[115]
	**MobileNetV3Large**	**0.9920**	**0.9840**	**1**	**0.9920**	**0.993**	**0.9850**	-	[116]

Interestingly, CNN does not consider multiclass classification as a confounding factor for diagnosis. Several studies have demonstrated the robustness of various neural network algorithms to non-binary classifications of different infectious diseases without a reduction in performance metrics [99,101,102,103,104,105,111,112]. As the dimensionality of data increases, clustering data points may become more challenging. Nonetheless, neural network algorithms remain largely unaffected in clustering data points based on disease categories. However, most studies have been evaluated on classification of COVID-19 and viral pneumonia cases [119]. Thus, more research will be necessary to validate the neural network algorithms’ performance when applied to other infectious diseases and symptoms.

Besides neural networks, ensemble methods that combine multiple ML algorithms have also demonstrated promise in diagnostic imaging. Traditionally, ensemble methods combine multiple supervised algorithms, such as LR, SVM, and XGBoost. However, two imaging studies in Table 2 demonstrate the utility of using Sequential Ensemble Learning—which is the consecutive deployment of feature selection algorithms to label the data followed by classification algorithms to classify the data [95,108]. With an increasing number of ML techniques used in classifying imaging data, future prospective studies that compare accuracy, sensitivity, and specificity between the different ML algorithms will provide essential insights into the most reliable methods for diagnostic image classification.

#### 3.5.2. Clinical Signs and Symptoms

Another attractive avenue for ML applications is in infectious disease diagnosis based on typical clinical signs [120,121,122,123,124,125,126,127] and self-reported symptoms [128,129]. Table 3 provides an overview of the critical studies that have used ML-based architecture in infectious disease diagnosis based on clinical signs and symptoms. Despite the promise of RF as a model for classification tasks, missing data from clinical studies can adversely impact the performance of RF [126], as RF is currently unable to manage and handle missing data effectively [130]. On the other hand, the ability of GBMs to impute missing data, including XGBoost, could be more suitable for clinical diagnosis based on symptoms and clinical parameters. This is evident in the good performance of GBMs when used alone [121,122,124] or when used in an ensemble with other models [120]. XGBoost has shown promise in the differential diagnosis of dengue compared to acute febrile illnesses when seasonality and prevalence of the disease are also considered [124]. Of note, the performance of ML decreases dramatically with self-reported symptoms [128], highlighting the importance of clinicians in providing quality assessments of clinical signs and symptoms for better diagnosis. Despite the models’ promise in helping clinicians to make better and faster diagnoses, clinician oversight on the final diagnosis is critical due to its implications on patient health, and the success of subsequent treatments. The role of ML and AI will not be to replace clinicians but rather to complement them.

**Table 3:** Overview of studies using ML-based architectures in infectious disease diagnosis for clinical signs and symptoms. A dash in a cell refers to metrics that are unreported by the study. The best-performing architectures in each category are in bold.

Category	Architecture	Accuracy	Precision/PPV	Recall/ Sensitivity	F1-Score	AUROC	Specificity	MCC	Reference
Clinical Signs	**GBM Ensemble + SHAP**	**0.96**	**0.94**	**0.95**	**0.94**	**0.98**	-	-	[120]
	XGBoost	-	-	0.819	-	0.97	0.979	-	[121]
	DNN Multi-Layer Perceptron	0.86	-	0.93	-	0.95	0.81	-	[125]
	XGBoost	0.822	-	0.797	-	0.905	0.845	-	[122]
	DNN Multi-Layer Perceptron	-	0.94	0.91	0.92	-	-	-	[127]
	RF	0.827	0.575	0.339	0.427	0.785	0.941	-	[126]
	XGBoost	-	0.73	0.56	-	0.86	0.92	-	[124]
	RF, LR, SVM, Multi-Layered Perceptron, XGBoost, AdaBoost Ensemble	-	0.29	0.93	-	0.91	0.64	-	[123]
Symptoms	Boosted LR	0.57	0.64	0.35	0.43	-	0.80	0.15	[129]
	**LR and Minority Data Upsampling**	**0.73**	**0.25**	**0.60**	**0.35**	**0.68**	**0.75**	**0.25**	[128]

Another critical consideration for ML models developed to diagnose infection from clinical signs is whether the ordered parameters are commonly included in routine clinical tests. While included parameters, such as granulocytes (neutrophils, basophils, and eosinophils) or erythrocytes (red blood cells), are commonly measured, disease-specific biomarkers may not be performed. For instance, the Non-Structural Protein 1 (NS1) antigen test to diagnose dengue infection may not be routinely tested for all patients unless dengue is suspected [131]. Therein lies the possibility of introducing selection bias when selected ML parameters are included retrospectively. Thus, the future development of models should focus on the standard test parameters and clinical signs for diagnosis, followed by reinforcement learning and model finetuning to recommend additional parameters for inclusion.

#### 3.5.3. Unstructured Text Classification

Unstructured text is ubiquitous in clinical notes where clinicians narrate detailed patient condition observations upon consultation. Some attempts have been made to use Large Language Models (LLMs) to interpret clinical signs and diagnoses. LLMs use a transformer architecture within Neural Networks, integrating supervised learning and Reinforcement Learning from Human Feedback (RLHF) for model finetuning. While the lack of interpretability poses a challenge to the implementation of LLMs, which is crucial to clinician understanding of infectious diseases and diseases in general [132], a study by Savage et al. reveals the potential for interpretability in LLMs for diagnostic applications by mimicking the chain of thoughts adopted by domain specialists [133]. Within the scope of infectious diseases, a study by Cheng et al. evaluates the potential applications of LLMs, like ChatGPT 3.5, within infectious disease diagnosis [134]. In the other study by Chiu et al., the authors demonstrated that LLMs can show interpretative reasoning in solving diagnostically challenging multimodal cases [135]. Future studies that compare the performance of LLMs in predicting ‘ground truth’ diagnoses of infectious diseases will be essential to demonstrate these models’ accuracy, sensitivity, and specificity in clinical diagnosis. The newly emerging GPT-4o architecture released by OpenAI on 13 May 2024 also offers opportunities for more clinical research to leverage LLMs in clinical diagnosis.

### 3.6. Roles of AI and ML Models in Clinical Prognosis

The goal of clinical prognosis is to be able to identify patients who are at risk of progressing to severe disease, which can be life-threatening, particularly in the elderly and young children. In the early phase of the disease, many patients exhibit clinical signs and symptoms of discomfort. The role of prognosis is thus to identify the early warning signs and symptoms that can reliably predict whether the infected individual will progress to severe disease and facilitate triage of patients, which is critical in settings where intensive care services may be limited [136].

The contribution of ML in the prognosis of infectious diseases has been focused on identifying clinical biomarkers that can predict disease progression [137,138,139,140,141,142,143,144,145,146,147,148,149,150,151,152,153,154,155]. Tree-based [139,140,141,145,149,150,153,154] and ensemble [138,148,155] approaches appear popular for identifying and ranking the relative contribution of various clinical biomarkers toward severe disease. On the other hand, some developments have been made in using ML to identify salient gene and protein expression in severe disease across multiple patient cohorts or studies [156,157,158,159]. Table 4 summarizes the current approaches to ML in infectious disease prognosis. XGBoost and ensemble learning have shown considerable promise in predicting severe disease, as seen from the high metrics in Table 4. Furthermore, XGBoost appears to be the dominant model of choice for infectious disease prognosis, given the higher proportion of studies that utilize it. The best models for clinical biomarkers and gene expression are bolded in Table 4.

**Table 4:** Overview of studies using ML-based architectures for infectious disease prognosis. A dash in a cell refers to metrics that are unreported by the study. The best-performing architectures in each image category are in bold.

Category	Architecture	Accuracy	Precision/PPV	Recall/ Sensitivity	F1-Score	AUROC	Specificity	MCC	Reference
Clinical Biomarkers	SVM	0.903	-	-	-	-	-	-	[137]
	Ensemble (Bagging)	-	0.86	0.98	0.91	0.79	-	-	[138]
	XGBoost	0.73	-	0.66	-	0.79	0.85	-	[139]
	XGBoost + SHAP	-	0.29	0.64	-	0.85	0.91	-	[140]
	XGBoost	0.9602	0.9533	0.9613	0.9573	0.9603	0.9591	0.8520	[141]
	LightGBM + SHAP	0.754	0.792	0.816	0.802	0.847	0.764	-	[142]
	Variational Autoencoders	-	0.62	0.75	-	-	0.71	-	[143]
	DNN + SHAP	-	0.3765	0.869	-	0.937	0.867	-	[144]
	GBM + SHAP	0.79	0.21	0.85	-	0.89	0.79	-	[145]
	ANN Backpropagation	-	-	-	-	0.8768	-	-	[146]
	Transformer + DNN	0.918	0.914	0.916	0.913	0.96	-	-	[147]
	Ensemble (RF, LightGBM) + SHAP	-	0.79	0.53	-	0.86	0.93	0.53	[148]
	LASSO + XGBoost + SHAP	-	0.882	0.918	0.937	0.94	-	-	[149]
	XGBoost	-	-	0.929	-	0.80	0.385	-	[150]
	DT	0.98	-	1.0	0.93	0.99	-	-	[151]
	ANN + SHAP	0.7523	-	-	-	0.8324	-	-	[152]
	LightGBM + SHAP	0.882	0.271	0.861	0.629	0.934	0.883	-	[153]
	DNN-Encoders + XGBoost	0.8278	-	-	-	-	-	-	[154]
	**Ensemble (RF, LR, DT, KNN, AdaBoost, CatBoost, LightGBM, XGBoost)**	**0.95**	**0.96**	**-**	**0.95**	**0.98**	**-**	**0.89**	[155]
Gene and Pathway Identification	**XGBoost**	**-**	**0.209**	**0.864**	**-**	**0.94**	**0.797**	**-**	[156]
	SVM, RF, LASSO	-	-	-	-	-	-	-	[157]
	RF	-	-	-	-	0.889	-	-	[158]
	LASSO	-	-	-	-	0.98	-	-	[159]

Simplicity of tests is an essential factor in determining the feasibility of proposed ML models. While the consideration of large numbers of biomarkers will likely yield better accuracy in predicting severe disease progression [150,152]. It should be noted that such an approach may be unpractical in clinical settings due to cost and patient discomfort [160]. Therefore, ML models should strive to identify the most important measurements that can predict severe disease progression. An ideal prognostic tool should use a minimal number of measurements yet deliver results with high sensitivity and specificity.

Another important consideration will be balancing interpretability, operational costs, and model performance. For instance, using Neural Networks may identify a diverse range of measurements that may be helpful for prognosis. However, taking all measurements may not be practical when considering the costs of testing involved. Including more testing may also increase the time needed for prognosis, which may be impractical under epidemic or pandemic settings [145,148]. Finally, it is important to note that the set of measurements or biomarkers for prognosis may differ between different demographic groups. For instance, typical prognostic markers for sepsis, such as Quick Sepsis-related Organ Failure Assessment (qSOFA), have lower sensitivity and specificity in older patients. Approaches such as using Procalcitonin (PCT) as a biomarker may improve sepsis management through demographic-sensitive differential prognoses [161].

**Figure 4:**
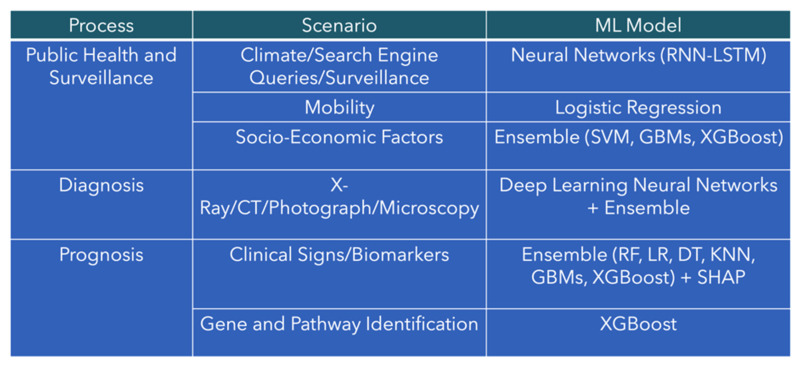
Summary of the most suitable ML models that can be used for infectious disease surveillance, diagnosis, and prognosis based on our literature review. Ensemble ML models demonstrate promise in multiple applications of infectious disease management. RNN-LSTM, Recurrent Neural Network–Long Short-Term Memory; SVM, Support Vector Machine; GBMs, Gradient Boosting Machines; XGBoost, eXtreme Gradient Boosting; RF, Random Forest; LR, Logistic Regression; DT, Decision Tree; KNN, k-Nearest Neighbor; SHAP, Shapley Additive exPlanations.

A summary of the best-known models for infectious disease surveillance, diagnosis, and prognosis is depicted in Figure 4. Despite ensemble learning architectures being commonly used as a part of currently developed ML models, the diversity of models currently available is an important aspect of note. While models can be used in tandem during deployment phases, there is no one-size-fits-all model for all scenarios. Each step in the infectious disease patient trajectory, as shown in Figure 4, necessitates different models which may be suited for its specific function.

## 4. Discussion

The infectious disease field has seen a notable surge in creating and deploying multiple models designed to address individual facets of infectious disease management, including public health, diagnosis, prognosis, and treatment. This review thus summarizes the current ML tools and algorithms that can be suitably used for infectious disease management.

While more ML algorithms have been developed for supervised learning, Explainable AI has demonstrated promise in achieving high accuracy in prediction. Explainability of models is vital to clinical practice as it instills greater trust in the models’ predictive capacity by both the clinician and the patient. A model’s predictions, regardless of its true accuracy, can see lower adoptions by clinicians if its output, decision making and differential diagnostic processes are untraceable. For example, suppose a model can diagnose dengue from a patient’s serum biomarkers. Trust in the dengue diagnosis is likely to be low if the model is unable to provide either the cutoff thresholds used, or reasoning for differential diagnosis of the case from related flaviviruses, such as Chikungunya or Zika. Therefore, model explainability is crucial to increase the propensity of change, i.e., model trust and adoption by both the clinician and the patient. Current examples of Explainable AI for other clinical applications exist, including predicting the efficacy of hematopoietic stem cell transplants in pediatric patients [162]. However, in many of these studies, external validation will be required to evaluate the robust performance of these ML models.

Furthermore, the lack of standardized guidelines for assessing models undermines the quality of model development. the existing guidelines, such as TRIPOD+AI [163] and CONSORT-AI [164], exist to encourage the transparent reporting of clinically based AI studies, but the guidelines are not always followed in a lot of cases. Additionally, besides deriving the most critical features or measurements required for infectious disease management, other important considerations should be pondered, such as operational costs, time, and integrability into clinical workflows.

Existing threats to validity in clinical settings include issues with data complexity and dataset biases [165]. The complexity of multimodal clinical data can arise from different arrangements, such as time-series data and clinical notes, and from various sources, such as imaging and biomarker data. This necessitates the importance of data cleaning, such as the removal of erroneous values and data imputation to prevent the introduction of confounders that distinguish populations rather than surfacing features of interest [165].

Biases in dataset curation could arise from improper representation of ethnicity and special patient populations, such as geriatric and pediatric patients. Strategies for external validation of ML or AI models within real-world settings include the deployment of models in multi-cohort and multi-demographic studies are increasingly important to ensure a low risk of bias. Some of such strategies propose for validating models on cohorts outside of the country in which the training and test dataset was developed in [126]. However, it is of note that not all models can be generalizable to all contexts. More thought needs to be given toward the claim of generalizability particularly with respect to the context of use and purpose of use. For example, if the ML or AI model is to be used in Germany, it should prove robust and generalizable to a wide range of German institutions as compared to Chinese institutions, given the context of use in both instances are different [166]. ML models should thus be adapted for different populations and demographic subgroups by customizing the approach to each population.

Diverse and representative datasets underpin not just a model’s generalizability but also serve to improve equitable health representation globally. LMICs are more likely to face a shortage of resources to collect large datasets of their population, leading to lower representation in the model development process. Various population subgroups around the world can contain idiosyncrasies, such as Single Nucleotide Polymorphisms or genomic variability, which necessitate dataset curation to identify such variations. This can result in clinical implications for treatment that may not be picked up if equitable access to such models were not provided. Therefore, providing LMICs with a voice to access new AI or ML models in the pipeline can increase equity in health representation.

A plausible solution for a model developer intending to develop a model within a new country market, then, could be to consult hospital authorities from the host country to understand the nuances of population demographics and clinical workflow. Although we provide a possible clinical workflow for the integration of ML in infectious disease on the subsequent page, we acknowledge that each country’s implementation setting, and consequently workflow, differ greatly. Additionally, resource-limited countries, for example, have computational resource constraints that limit the collection and processing of data. Therefore, the development of population-specific ML models in infectious disease must account for resource differences. Future studies should pay attention when training clinical ML models to identify the presence of dataset bias and mitigate it using methods such as variational autoencoders to overrepresent selected data subgroups [167]. Alternatively, imputation is a possible solution to dataset bias. Existing imputation methods, such as KNN imputation, have been identified to have a superior performance when handling cohort datasets [168].

### 4.1. Implications

For an ML or AI model to be effective in clinical settings, the model must obtain the trust of clinicians using easily traceable machine decision processes. Trust would mean clinicians can rely on the model to improve human oversight without actively monitoring the system’s outputs. The low interpretability of some models does not improve the performance of junior clinicians, who should be the primary beneficiaries of such systems due to less clinical experience [169]. Utilizing XAI and methods highlighting feature importance should be the primary ways to increase the transparency of the rationale behind classifier prediction [170]. Studies on XAI-informed ML by Chadaga et al. (2024) [155] and Boussina et al. (2024) [171] have good potential for clinical implementation due to a low risk of bias. Ideally, future studies attempting to develop clinically translatable ML models should be easy to use and not add unnecessary hindrances to already complicated patient management workflows. The aim of ML should be to complement clinician decision-making rather than replace clinicians.

Given the multimodal nature of infectious disease cases, employing ensemble learning to combine diverse parameters rather than examining parameters in isolation shows promise in replicating real-world clinical scenarios more effectively, increasing the chance of facilitating more seamless integration into clinical workflows. It is worth noting that five out of the nine best models in the categories within infectious disease management employ various forms of ensemble learning [95,108,111,120,155]. The promise of ensemble learning suggests that further research into ML in infectious diseases can explore the possibility of incorporating ensemble learning models with hard/soft voting systems. Moreover, public–private partnerships can enhance the customization of pre-developed ensemble learning models to fit into clinical decision support systems already available in hospitals to increase clinician familiarity and operability.

### 4.2. Limitations

There are some limitations in this review. As the review is intended to provide a broad overview of the current applications of ML and AI within infectious disease, the reported metrics in summary tables located in the respective sections do not report the context and limitations of studies in comprehensive detail. Due to the limited availability of public datasets, we were also not able to directly evaluate and compare the accuracy, sensitivity, and specificity of the ML models. In addition, we included only the literature published in English and from Google Scholar, PubMed, and ScienceDirect, so we may have missed out on the quality literature and its associated data emerging from other database sources, such as Web of Science or Scopus. Additionally, the review may have missed the relevant gray literature, such as preprints, dissertations, or government reports, not indexed in the databases. We also do not exclude the possibility of potentially missing out the literature that is relevant for ML based on the keywords used in our literature search. The rapid pace of advancement in AI and ML may cause some findings or recommendations in this review to become outdated.

Furthermore, this review assumes a disease-agnostic comparison, which assumes the comparable performance of models across various infectious disease modalities. Differences in pathogenesis, healthcare contexts, and the available existing data could limit the generalizability of model comparisons. Additionally, models which work well on a certain pathogenic modality (e.g., bacterial infections) should not be assumed to work in other modalities (e.g., viral, fungal infections). Importantly, further research can be carried out to test the effectiveness of the models mentioned in this review in specific disease subgroups to control for heterogeneity. Given that AI and ML models are evolving rapidly, we also acknowledge that our recommendations made in this study may not be relevant to the development of new AI and ML models in the future. Nonetheless, the selection of studies and sources in this review was made carefully to ensure a broad and representative understanding, and the review provides a vast landscape of the ML models employed in infectious disease management.

### 4.3. Future Directions

**Figure 5:**
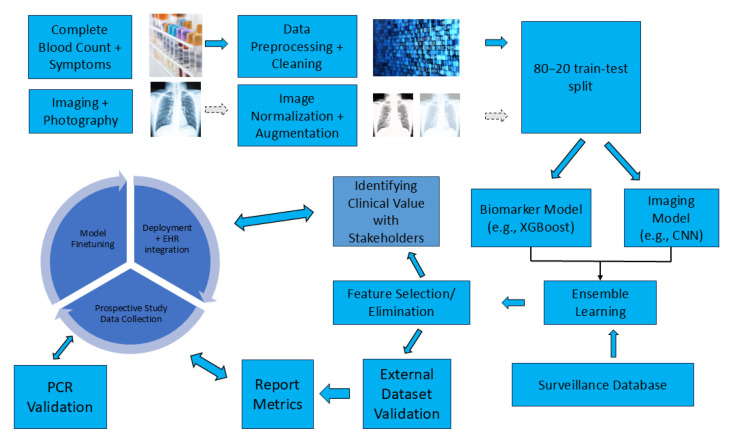
A possible workflow for clinical machine learning models in infectious diseases using information from this review.

Critical avenues for future research include applying translational clinical ML and a practical model evaluation within clinical settings. Operational frameworks on translational clinical ML have been developed which can be used in the infectious disease field [172]. Figure 5 illustrates a proposed workflow for clinical ML, emphasizing an iterative process that involves identifying the clinical value and significance of the model in collaboration with key stakeholders, including clinicians. The models in this review can be incorporated within such a framework to enhance their translational relevance.

Moreover, the potential for cloud-based storage combined with diagnostic methods can enhance the deployment of such ML models [120,155]. A combination of symptoms across disease modalities may help in clinical decision-making aided by ML [128]. Furthermore, a model that compares the performance of these ML models will help identify the best ML algorithms for deployment in public health and clinics. Model trials and integration into the diagnostic process commonly found in places where infectious disease patients have the highest contact, such as in infectious disease service wards, may result in clinically useful results. Additionally, a meta-analysis into the evolution and diversity of ML applications utilized in various aspects of infectious disease management as covered by this review can provide statistically informed insights into the direction of the field.

### 4.4. Challenges in AI and ML Implementation

While the implementation of AI and ML is promising in the field of infectious diseases, there are important ethical considerations that should be addressed. Importantly, ethical guidelines and data security must be established to prevent the misuse of patient data. Given the promise of using Explainable AI and ensemble learning in infectious disease management, and these algorithms are likely executed by skilled bioinformaticians, it is important to ensure that the patient identity is protected by anonymizing the data and encrypted to protect patient information. Finally, the implementation of AI and ML should not result in reduced supervision and training, which can negatively impact the competency of clinicians. Thus, the use of AI and ML should be used with constant monitoring, evaluation, and improvements to ensure accuracy and prevent over-reliance.

## 5. Conclusions

In this review, we provided a comprehensive review on the ML and AI tools used in infectious disease surveillance, clinical diagnosis, and prognosis. Among the different ML models, Explainable AI and ensemble learning have greater potential for broad clinical applications due to higher accuracy, transparency, and low risk of bias. The current ML and AI models can provide accurate prediction and outcomes, which can consequently improve infectious disease surveillance, diagnosis, and clinical prognosis. While the performance of these methods will have to be evaluated and validated in larger clinical cohorts, these findings encourage the deployment of ML and AI to complement clinicians and improve clinical decision-making.

## Data Availability

No new data were created.

## Abbreviations

The following abbreviations are used in this manuscript:

COVID-19	Coronavirus Disease 2019
LMICs	Low- and Middle-Income Countries
AI	Artificial Intelligence
ML	Machine Learning
XAI	Explainable AI
SHAP	Shapley Additive exPlanations
Grad-CAM	Gradient-weighted Class Activation Mapping
CNNs	Convolutional Neural Networks
CT	Computed Tomography
RF	Random Forest
AUROC	Area Under Receiver Operating Characteristic
MCC	Matthew’s Correlation Coefficient
CC	Chief Complaint
PCR	Polymerase Chain Reaction
FN	False Negative
TN	True Negative
FP	False Positive
TP	True Positive
EIDs	Emerging Infectious Diseases
HAIs	Hospital Acquired Infections
SARS-CoV-2	Severe Acute Respiratory Syndrome-Coronavirus 2
RBD	Receptor Binding Domain
SEQs	Search Engine Queries
SVM	Support Vector Machine
RNNs	Recurrent Neural Networks
LSTM-ATT	Attention-Based Long-Short Term Memory
SEIR	Susceptible-Exposed-Infected-Recovered
LR	Logistic Regression
KNN	k-Nearest Neighbor
XGBoost	eXtreme Gradient Boost
NLP	Natural Language Processing
PCC	Pearson’s Correlation Coefficient
DNNs	Deep Neural Networks
GBMs	Gradient Boosting Machines
LASSO	Least Absolute Shrinkage and Selection Operator
ARI	Acute Respiratory Infection
DTs	Decision Trees
RMSE	Root Mean Squared Error
FDA	Food and Drug Administration
NS1	Non-Structural Protein 1
LLMs	Large Language Models
QSOFA	Quick Sepsis-related Organ Failure Assessment
PCT	Procalcitonin
